# Understanding prosthesis patient mismatch in aortic valve replacement: from pitfalls to clinical impact

**DOI:** 10.1007/s12928-026-01331-w

**Published:** 2026-08-07

**Authors:** Akihiro Tobe, Patrick W. Serruys, Yoshinobu Onuma, Christian Juhl Terkelsen, Mads Jønsson Andersen, Michele Gallazzi, Angela McInerney, Scot Garg, Jose Luis Pomar

**Affiliations:** 1 CORRIB Research Centre for Advanced Imaging and Core laboratory, University of Galway, Galway, Ireland; 2 Department of Cardiology, Aarhus University Hospital, Aarhus, Denmark; 3 Department of Medical and Surgical Sciences, Faculty of Medicine and Surgery, School of Cardiovascular Diseases, University of Milan, Milan, Italy; 4 Department of Cardiology, University Hospital Galway, Galway, Ireland; 5 Department of Cardiology, Royal Blackburn Hospital, Blackburn, UK; 6 School of Medicine, University of Central Lancashire, Preston, UK; 7 The Cardiovascular Institute Hospital Clinic & University of Barcelona, Barcelona, Spain

**Keywords:** Prosthesis patient mismatch, Transcatheter aortic valve replacement, Surgical aortic valve replacement, Aortic stenosis, Fitting index

## Abstract

Almost half a century ago, cardiac surgeons introduced the concept of prosthesis–patient mismatch (PPM), which was defined by indexing the effective orifice area (EOA) to body surface area. Although PPM has demonstrated strong prognostic value following surgical aortic valve replacement (SAVR), its significance in transcatheter aortic valve replacement (TAVR) remains uncertain. In this review, we revisit the concept of PPM and explore the methodological and clinical challenges in its application. Special focus is given to the limitations in interpreting EOA and indexed EOA (EOAi), including flow dependency and imaging variability. We also review the literature evaluating the association between PPM and clinical outcomes after SAVR and TAVR. Finally, we discuss a potential role of alternative metric such as the fitting index.

**Graphical Abstract:**

**Figure:**
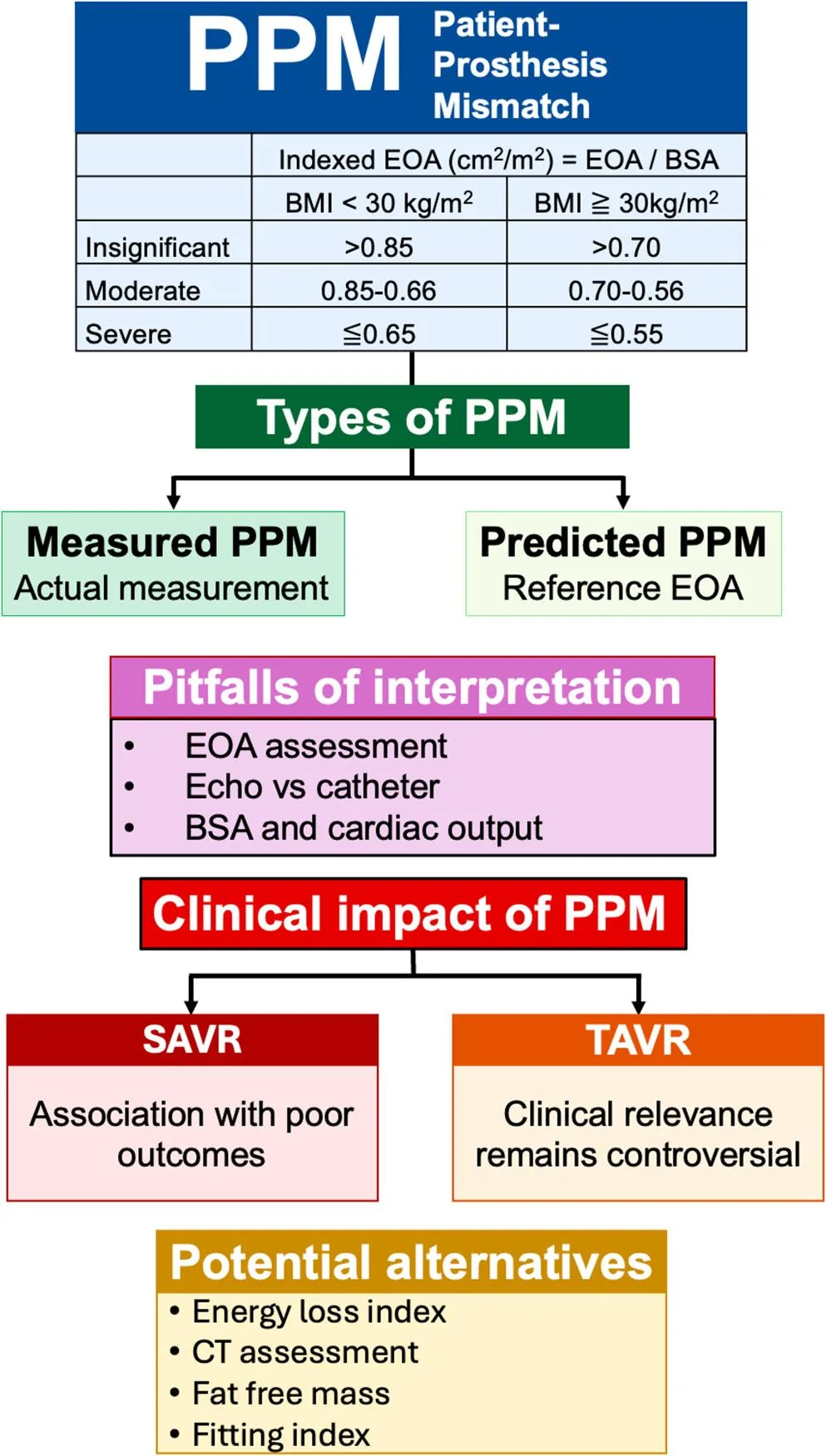

**Supplementary Information:**

The online version contains supplementary material available at https://doi.org/10.1007/s12928-026-01331-w.

## Concept of PPM

Prosthesis–patient mismatch (PPM) is defined as an effective orifice area (EOA) of an implanted prosthetic valve that is too small relative to the patient’s body size [1]. This concept was first proposed by the cardiovascular surgeon Rahimtoola SH in 1978 in the context of surgical aortic valve replacements (SAVR) [2]. The normal aortic valve area is typically 3–4 cm² ; however, prosthetic valves rarely achieve this [3,4]. Therefore, whilst a degree of PPM occurs in nearly all cases, it is usually clinically insignificant, unless the mismatch is substantial.

**Fig. 1:**
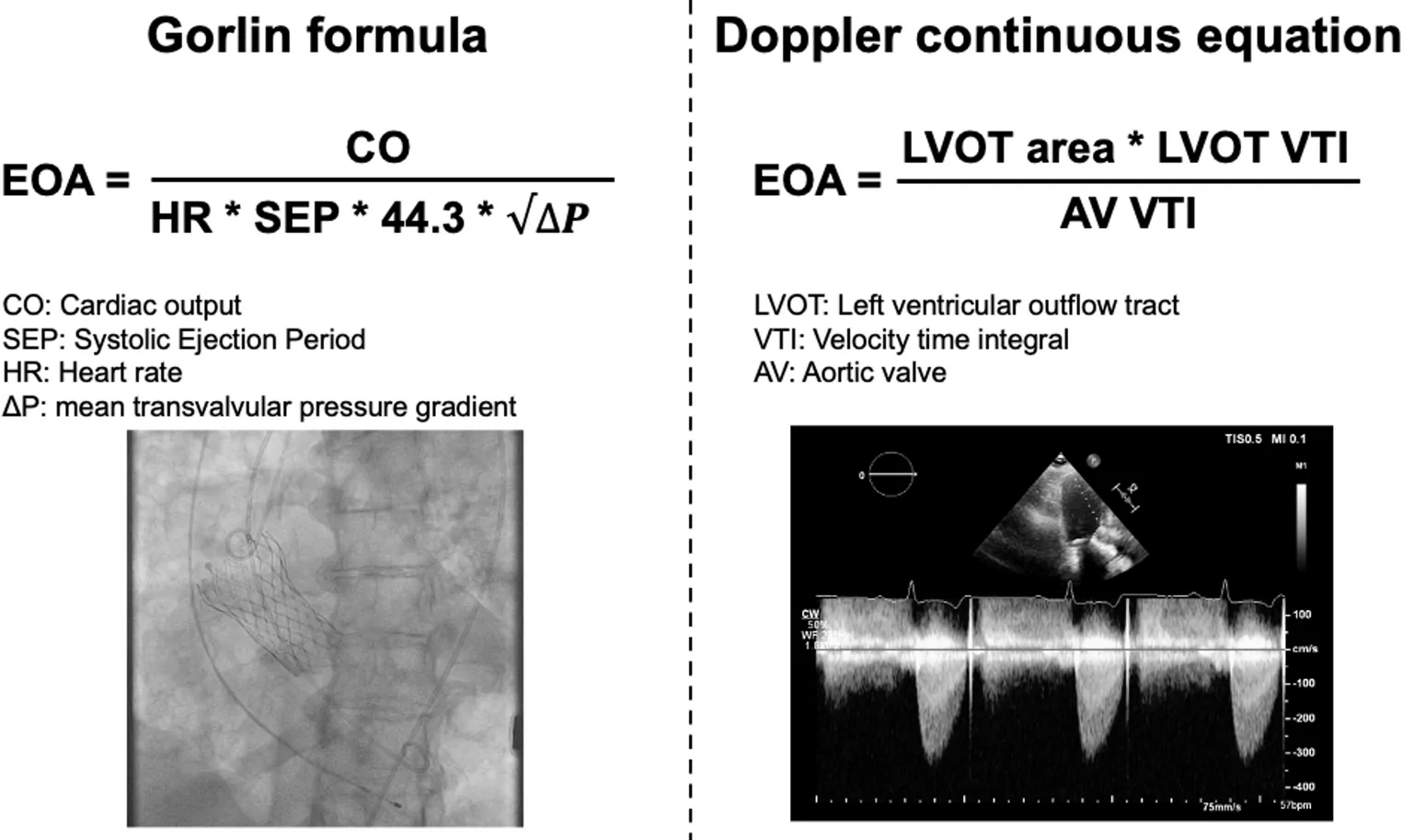
Gorlin formula and Doppler continuous equation. EOA: effective orifice area

The EOA represents the functional area through which blood actually flows across the valve, as opposed to the geometric valve orifice area. It is determined using either invasive hemodynamic assessment or Doppler echocardiography. Before echocardiography and Doppler assessment were established, the EOA was derived invasively using Gorlin’s formula (Fig. 1), with cardiac output measured by dye dilution (cardiogreen) or thermodilution, and pressure gradients obtained via catheterization [5]. In contemporary practice, the EOA is usually calculated by Doppler echocardiography (Fig. 1).

### Definition of PPM

PPM is assessed by indexing the EOA to body surface area (BSA), as the absolute EOA does not account for the impact of body size on cardiac output requirements: the indexed EOA (EOAi) = EOA/BSA [6]. Previously, many studies used cut-off values of 0.85–0.66 cm^2^/m^2^ for moderate PPM and ≤ 0.65 cm^2^/m^2^ for severe PPM [7], however, in obese patients, the BSA may be disproportionately high relative to their actual metabolic and cardiac output needs. Therefore, applying the same EOAi cut-off values for obese patients may lead to the severity of PPM being overestimated. In the Valve Academic Research Consortium (VARC)-3 lower EOAi cut-off values (0.70–0.56 cm^2^/m^2^ for moderate PPM and ≤ 0.55 cm^2^/m^2^ for severe PPM) are recommended for obese patients (BMI ≥ 30 kg/m^2^) (Table 1) [8].

**Table 1:** Criteria of PPM

	Indexed EOA (cm^2^/m^2^) = EOA / BSA	
	BMI < 30 kg/m^2^	BMI ≥ 30 kg/m^2^
Insignificant	>0.85	>0.70
Moderate	0.85-0.66	0.70-0.56
Severe	≤0.65	≤0.55

BMI=body mass index, BSA=body surface area, EOA=effective orifice area, PPM=prosthesis patient mismatch

### Mechanism of PPM

PPM can occur for several reasons. Like placing a tube within a tube, the implanted prosthesis must be smaller to fit inside the native annulus, thereby reducing the EOA. This is particularly problematic in patients whose annular dimensions are already small relative to their body size, unless a surgical aortic root enlargement (a procedure increasingly performed in recent years) is also performed [9,10]. This issue is especially pronounced with stented bioprosthetic SAVR, which require a sewing ring for fixation – this component occupies space and further reduces the EOA. The thickness of the sewing ring also accounts for the discrepancy between the labeled valve size and the stent’s internal diameter. In addition, the mounting position of the leaflets, whether the leaflets are mounted internally or externally, can cause further variation between the stent’s internal diameter and the true internal diameter [11]. Valves with internally mounted leaflets tend to have smaller true internal diameters and thus a higher risk of PPM [12]. Finally, BSA is the denominator for determining the EOAi.

Transcatheter aortic valve replacement (TAVR) is generally associated with a lower risk of PPM compared to SAV R[13]. This is because TAVR valves are anchored by radial force rather than sutured, and thus lack a sewing ring, resulting in a larger EOA. In echocardiographic assessment, a supra-annular design is associated with a higher EOA than an intra-annular design; however, this may not hold true under invasive measurement (as described later).

Table 2 shows how body size influences PPM, comparing the incidence of PPM between US and Japanese patients treated with TAVR using the Sapien 3 Ultra Resilia [14,15]. Although both populations had similar EOA values post-TAVR, the larger body size in the U.S. cohort resulted in smaller EOAi values and a correspondingly higher incidence of severe PPM.

**Table 2:** Studies of Sapien 3 Ultra Resilia in TAVR registries in U.S and Japan

	TVT registry (U.S.)^14^ *N*=10,312	OCEAN-TAVI registry (Japan)^15^ *N*=618
BMI, kg/m^2^	29.7±9.6	22.2±3.7
BSA, m^2^	NA	1.49±0.18
**EOA**,** cm**^**2**^	**1.9±0.6**	**1.86±0.47**
**EOAi**,** cm**^**2**^**/m**^**2**^	**0.9±0.3**	**1.26±0.31**
Moderate PPM, %	NA	5.2%
**Severe PPM**,** n (%)**	**6.2%**	**0.5%**

BMI=body mass index, BSA=body surface area, EOA=effective orifice area, EOAi=indexed effective orifice area, NA=not available, PPM=prosthesis patient mismatch, TAVR=transcatheter aortic valve replacement

PPM is a functional hemodynamic baseline abnormality, rather than a structural flaw in the prosthetic valve. It can occur even when the implanted valve is functioning normally, and in theory, is present immediately post-op. However, it is important to distinguish this post-procedural baseline from “acquired” PPM at follow-up, which results from a reduced EOA due to progressive valve deterioration over time. In such cases, an underlying cause such as structural valve dysfunction (leaflet fibrosis, calcification, stent deformation, etc.), as well as thrombosis or endocarditis, may be responsible for the temporal and dynamic alteration of PPM [16]. Measurement factors, such as image quality, measurement variability, or changes in flow status, may also contribute to the apparent development of acquired PPM. Additionally, changes in body weight can also affect PPM classification. Even when the intrinsic valve function remains unchanged, weight loss may increase EOAi and potentially reduce the severity of PPM. This phenomenon may also occur in patients receiving weight-loss drugs, such as GLP-1 receptor agonists.

## Challenges in the assessment of PPM: EOA

Most studies currently assess the EOA using Doppler echocardiography and the following formula: EOA (cm^2^) = left ventricular outflow tract (LVOT) Area × LVOT velocity-time integral (VTI) / aortic valve VTI. The primary advantage of echocardiographic assessment is that it is non-invasive and widely accessible; however, it has several limitations. First, as with all imaging modalities, poor image quality may hinder accurate measurements. Second, measurements must be performed according to established guidelines. For example, the LVOT diameter should be measured outer edge–to–outer edge at the lower (ventricular) end of the valve stent, whilst the LVOT velocity-time integral (VTI) should be obtained just proximal to the stent frame [1,17]. Even small deviations from these recommendations can lead to substantial differences in the calculated EOA. For example, if the LVOT diameter is measured inner edge–to–inner edge, the derived EOA may be significantly underestimated. In addition, incorrect placement of the pulsed-wave Doppler sample volume within the zone of flow acceleration, rather than in the true LVOT just proximal to the stent frame, may lead to overestimation of LVOT VTI and further overestimation of the calculated EOA.

Third, the LVOT area is typically estimated using the formula LVOT Area = π × (LVOT diameter/2)², which assumes that the LVOT is circular; however, it is well established that it is elliptical. Contrast-enhanced computed tomography (CT) can assess the LVOT area more accurately and has been shown to significantly increase it, and thereby reduce the incidence of PPM compared to transthoracic echocardiography (TTE) [18].

Fourth, velocity-time-integral (VTI) assessment using Doppler echocardiography is dependent on flow status. Prosthetic leaflet opening may be incomplete in patients with low-flow states, potentially leading to an underestimation of EOA and, consequently, an overestimation of PPM severity. This phenomenon parallels the concept of pseudo-severe aortic stenosis in patients with low-flow, low-gradient native aortic stenosis. Fifth, due to pressure recovery, Doppler- and catheter-derived pressure gradients may differ, as described later.

### Two types of PPM: measured and predicted

There are two types of PPM: measured and predicted. Measured PPM is based on the actual EOAi after valve replacement. In contrast, predicted PPM is calculated using reference values of the EOA provided by manufacturers or published data [19]. In the setting of TAVR, Hahn et al. reported reference EOA values for the Sapien/Sapien XT/Sapien 3 valves using data from the PARTNER 1, PARTNER 2 A, PARTNER 2B, and PARTNER S3 studies, and for the CoreValve/Evolut R valves using data from the CoreValve US Pivotal Extreme Risk and High Risk trials and the Medtronic CoreValve Evolut R United States IDE Clinical Study [20].

There are several important distinctions between the two approaches.

First, measured PPM can only be assessed after the procedure, whereas predicted PPM can be estimated before the procedure, enabling the physician to consider potential PPM during valve selection.

Second, TTE assessment in the early postoperative phase is often limited by suboptimal imaging windows, especially post-SAVR. Therefore, in clinical practice, the use of predicted PPM may offer a more practical approach in SAVR patients.

Third, as described earlier, the measurement of EOA depends on image quality, technical errors/variations, and flow status. In contrast, predicted PPM, which uses reference EOA derived from standardized assessments, partly overcomes these limitations. However, this advantage is contingent upon the assumption that the reference EOA values have been obtained under appropriate conditions, using correct methodology and normal flow states. In TAVR, reference EOA values used for predicted PPM are typically derived from standardized datasets analyzed in core laboratories,^20^ whereas in SAVR they are often based on more heterogeneous historical sources. Thus, predicted EOA in SAVR and TAVR may not be directly analogous constructs. Importantly, while this methodological standardization represents a key advantage of predicted PPM in TAVR, the reference EOA is derived from Doppler measurements and therefore still subject to the inherent limitations.

The clinical relevance and prognostic implications of measured and predicted PPM will be discussed separately in subsequent sections.

### Pressure recovery: echocardiographic versus catheter-based assessment of EOA

During systole, blood flow across a prosthetic valve accelerates and converges just distal to the valve in a narrow region known as the vena contracta. At this point, flow velocity peaks, pressure reaches its lowest point, and potential energy is converted into kinetic energy. Some of this energy is lost as heat and friction, while another portion contributes to turbulent flow. These factors result in a pressure gradient, which is captured by Doppler echocardiography.

**Fig. 2:**
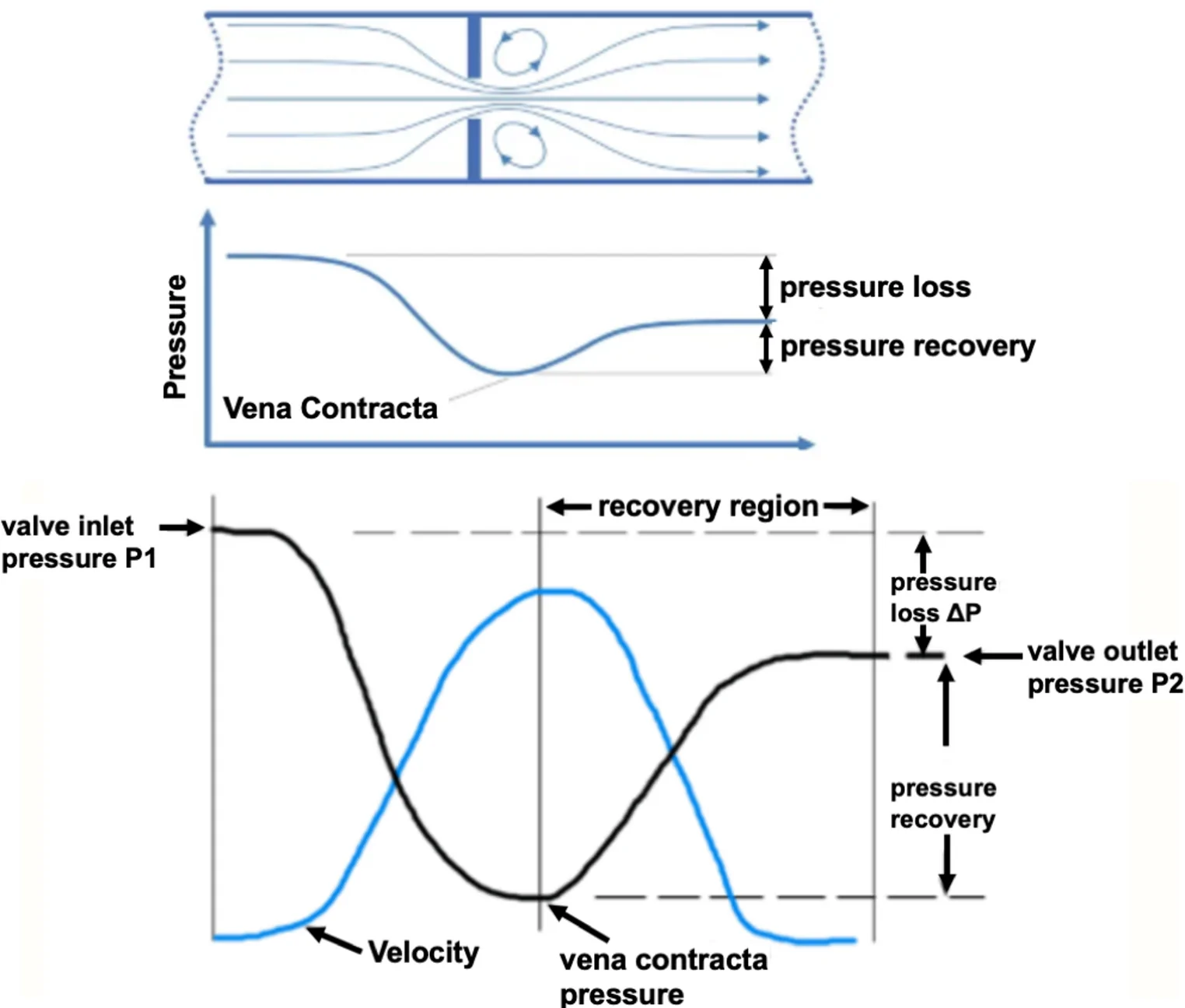
Vena contracta and Pressure recovery. The figure was quoted from https://physics.stackexchange.com/questions/717442/pressure-recovery-what-is-it-and-why-do-we-want-it with modification

Further downstream in the ascending aorta, turbulent flow gradually transitions to more laminar flow, allowing partial pressure recovery (Fig. 2) [21]. Catheter-based pressure measurements are typically obtained in this downstream region, after pressure recovery has occurred, and therefore, aortic pressure measured by catheterization is higher than the pressure at the vena contracta. Consequently, the pressure gradient measured by catheterization is often lower – and sometimes markedly different – than that measured by echocardiography [22].

**Fig. 3:**
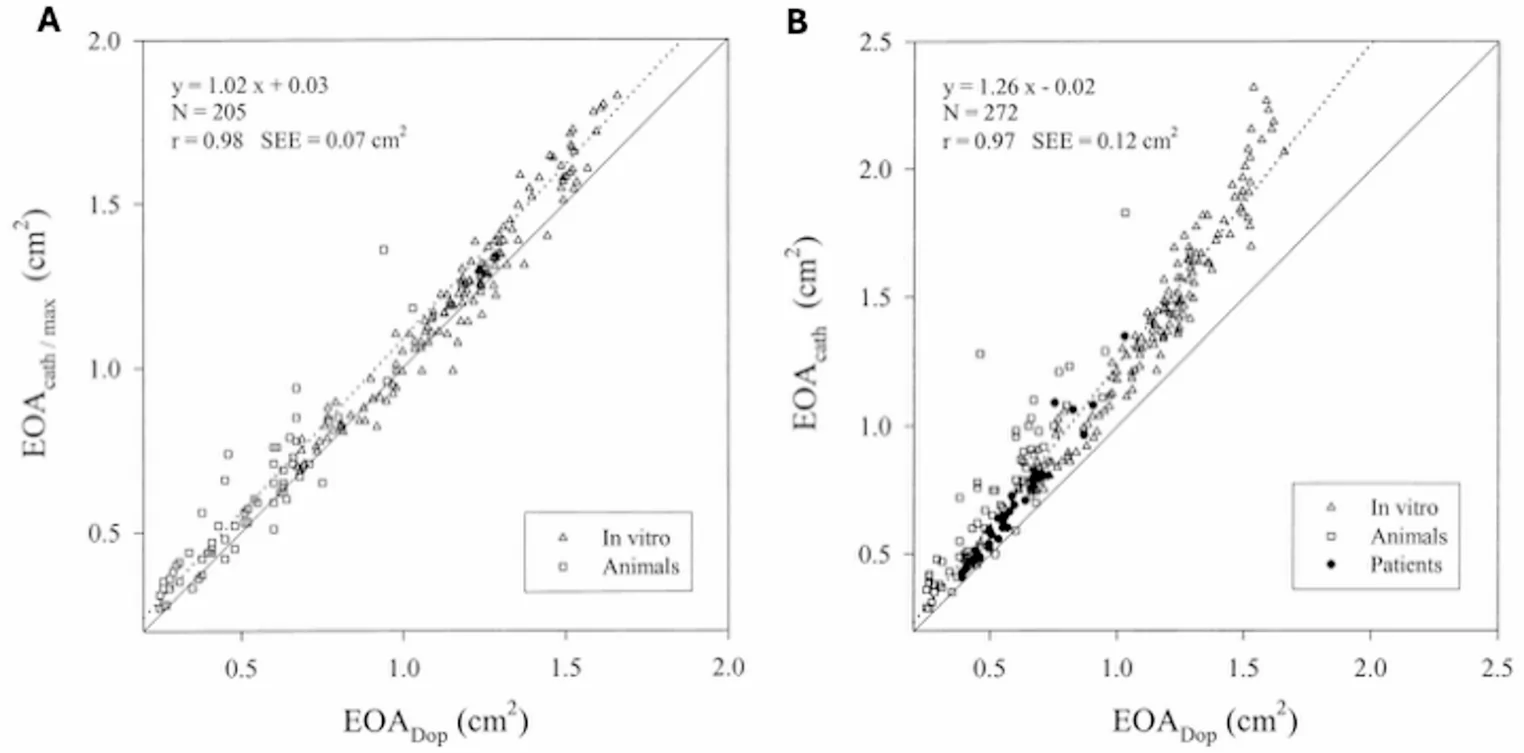
EOA measurement by Doppler vs. catheter. A: EOA (Doppler) vs. EOA (catheter at vena contracta). B: EOA (Doppler) vs. EOA (catheter after pressure recovery). Figures are quoted from Garcia D et al.^23^

In 2003, Garcia et al. compared Doppler-derived and catheter-derived measurements in vitro, in animal models, and in human subjects. They found that the catheter-derived EOA, calculated from pressure measured at the vena contracta (i.e., the point of lowest pressure), correlated better with Doppler-derived EOA, than when pressures obtained at the level of pressure recovery were used. Furthermore, EOAs calculated from pressure recovery levels were systematically larger than those derived by Doppler (Fig. 3) [23].

Recent studies assessing pressure gradients following TAVR confirmed this discrepancy, demonstrating that catheter-derived gradients at the level of pressure recovery were consistently lower than those measured by echocardiography. Of note, this discrepancy is more pronounced in supra-annular self-expanding THVs than in intra-annular balloon-expandable THVs [24–27]. Hatoum H et al. conducted an experimental study measuring pressure gradients at the vena contracta and the pressure recovery zone, comparing a 23 mm Sapien 3 with a 26 mm Evolut valve. Although the gradient at the vena contracta was higher with the Sapien 3, the gradient after pressure recovery was significantly lower compared with Evolut [25]. Van den Dorpel MMP et al. reported that it was not the echocardiographic but the catheter-derived gradients that were associated with mortality [26].

Deciding whether echocardiographic or catheter-based gradients should take priority remains a major ongoing topic of discussion.

### Limitations of the gorlin formula

Before the advent of Doppler echocardiography, the Gorlin formula based on invasive hemodynamics was the standard method for assessing aortic stenosis, and Doppler-based assessment has evolved in part from these principles. However, the Gorlin formula is also primarily dependent on the mean pressure gradient and cardiac output, with ejection time and heart rate exerting a modest impact on the calculation of aortic valve area (AVA) [28]. Importantly, cardiac output itself is estimated (e.g., by thermodilution), which may introduce additional measurement variability and affect the calculated AVA.

**Supplementary Fig. 1** illustrates the relationship between the mean pressure gradient and the AVA derived from the Gorlin formula, assuming constant heart rate and ejection time. At very low mean gradients, the calculated AVA may become disproportionately large—sometimes reaching physiologically implausible values. This phenomenon is often observed after successful TAVR procedures, where gradients are rarely > 10 mmHg [29]. However, the threshold at which low gradients begin to meaningfully distort the calculated AVA remains uncertain and should be interpreted with caution. Despite these limitations, invasive assessment retains important complementary value. In particular, pressure gradients are directly measured during catheterization, unlike Doppler-based estimates. Although the calculated AVA may become less reliable under low-gradient conditions, a low directly measured pressure gradient itself argues against the presence of clinically significant obstruction. Therefore, invasive hemodynamic assessment should be considered complementary to Doppler-based evaluation, rather than viewed solely in terms of its limitations.

## Challenges in assessment of PPM: Is body surface area an accurate surrogate for cardiac output?

Indexing EOA by BSA assumes that cardiac output requirements are proportional to BSA; however, whether this applies to patients undergoing AVR remains uncertain. In the PERIGON trial, Vriesendorp MD et al. evaluated 744 SAVR patients with normal LV function and found only a weak correlation between BSA and cardiac output (cardiac output = 1.5*BSA + 1.9, *r* = 0.29) [30]. Furthermore, while larger BSA increased the likelihood of being labelled with PPM, it was not associated with an increased risk of having hemodynamic obstruction (mean gradient ≥ 20 mmHg and/or a Doppler velocity index < 0.35). The authors thus concluded that the current definition of PPM may systematically overestimate hemodynamic obstruction in patients with larger BSA, suggesting caution in using indexed EOA in this subgroup.

## Clinical impact of PPM

### Impact of PPM in SAVR

In SAVR, predicted PPM has often been used to investigate the incidence and impact of PPM, with numerous studies confirming its negative impact on clinical outcomes (Table 3) [31–34]. PPM is associated with mortality, and other adverse clinical outcomes, including heart failure hospitalizations, reduced improvement in quality-of-life and functional status, and less regression in left ventricular mass [13].

**Table 3:** Selected studies reporting clinical outcomes of PPM after SAVR

First Author	Database	Year	*N* of patients	treatment	Type of PPM	Criteria of PPM	Incidence of PPM	Annular enlargement	FU	Mortality
Mannacio V [31]	Multi center	2017	3,082	SAVR with a stented bioprosthesis	Measured PPM	#1	Moderate: 70%	0% (excluded)	10 years	Moderate vs. severe (excluding operative mortality): 43.1% vs. 57.1%, *p*=0.006
							Severe: 8%			
Fallon JM [32]	STS registry	2018	59,779	Isolated SAVR	Predicted PPM	#1	Moderate: 54%	3.2%	10 years	Moderate vs. none: 57% vs. 54% (adjusted HR 1.08, CI 1.05-1.12)
							Severe: 11%			Severe vs. none: 65% vs. 54% (adjusted HR 1.32, CI 1.25-1.39)
										Severe vs. moderate: 65% vs. 57% (adjusted HR 1.22, CI 1.16 -1.28)
Dahlbacka S [33]	FinnValve registry	2021	4,097	SAVR with a stented bioprosthesis	Predicted PPM	#1	Moderate: 39%	NA	5 years	Moderate vs. none: 20.8% vs. 14.9%, not significant
							Severe: 7%			Severe vs. none: 29.3% vs. 14.9%, adjusted HR 1.72 (CI 1.08-2.76, *p*=0.02)
Dismorr M [34]	SWEDEHEART registry	2023	16,423	SAVR with a stented bioprosthesis	Predicted PPM	#2	Moderate: 52%	NA	10 years	Moderate vs. non: 45% vs. 43%, difference 1.7% (CI 0.1-3.3%)
							Severe: 3%			Severe vs. none: 48% vs. 43%, difference 4.6% (CI 0.7-8.5%)

#1: none (>0.85 cm^2^/m^2^), moderate (0.85-0.65 cm^2^/m^2^), and severe (≤0.65 cm^2^/m^2^)

#2 (VARC3 criteria): If BMI is <30 kg/m^2^, none (>0.85 cm^2^/m^2^), moderate (0.85-0.66 cm^2^/m^2^), and severe (≤0.65 cm^2^/m^2^). If BMI is ≧30 kg/m2, none (>0.70 cm^2^/m^2^), moderate (0.70-0.55 cm^2^/m^2^), and severe (≤0.55 cm^2^/m^2^)

CI=confidence interval, HR=hazard ratio, NA=not available, PPM=prosthesis patient mismatch, SAVR=surgical aortic valve replacement

A report from the STS registry (*n* = 59,779) of patients undergoing isolated SAVR demonstrated a stepwise increase in 10-year mortality with increasing severity of predicted PPM [32]. The FinnValve registry (*n* = 4,097) reported that severe predicted PPM—but not moderate predicted PPM—following SAVR with stented bioprostheses was associated with increased 5-year mortality compared to patients without PPM [33]. Data from the SWEDEHEART and other national Swedish registries (*n* = 16,423) also demonstrated a stepwise increase in long-term all-cause mortality over 10 years with increasing predicted PPM grade in patients undergoing bioprosthetic SAVR. However, the absolute risk differences in patients with moderate predicted PPM were small. The authors concluded that the clinical significance of moderate predicted PPM might be negligible and potentially outweighed by the risks associated with preventive strategies [34].

Several meta-analyses have also reported that PPM after SAVR is associated with higher mortality (Table 4) [35–39]. Although severe PPM has consistently been linked to increased mortality, the prognostic impact of moderate PPM has varied across studies. Sá et al. conducted a meta-analysis investigating the impact of PPM after SAVR using reconstructed survival curve data from 122,989 patients. Both moderate and severe PPM were associated with increased long-term mortality and heart failure hospitalization. The meta-analysis included studies that assessed the EOA using different methods: in vitro measurements provided by manufacturers, in vivo values reported in published studies, and actual measurements from Doppler echocardiography. Across all methods, ≥moderate PPM was consistently associated with worse survival, with the most pronounced survival differences observed in studies using Doppler echocardiography-derived EOA [39].

**Table 4:** Selected meta-analyses reporting clinical outcomes of PPM after SAVR/TAVR

First Author	Year	*N* of studies	*N* of patients	SAVR/TAVR	Incidence of PPM	Mortality
Head SJ [35]	2012	34	27,186	SAVR only	Any: 44.2%	Any PPM vs. none: HR 1.34 (1.18 – 1.51)
					Moderate: 34.2%	Moderate vs. none: HR 1.19 (1.07 – 1.33)
					Severe: 9.8%	Severe vs. none: HR 1.84 (1.38 – 2.45)
Takagi H [36]	2012	33	25,909	SAVR only	Any: 32.5%	Any PPM vs. none: HR 1.31 (1.16 – 1.48), *p*<0.0001
					Moderate: 43.8%	Moderate vs. none: HR 0.99 (0.92 – 1.07), *p*=0.78
					Severe: 10.9%	Severe vs. none: HR 1.27 (1.11 – 1.46), *p*=0.0008
Chen J [37]	2014	14	14,874	SAVR only	–	Any PPM vs. none (10 years): HR 1.52 (1.26 – 1.84)
						Moderate PPM vs. no PPM: Adjusted HR 0.96 (0.86 – 1.07)
						Severe PPM vs. no PPM: Adjusted HR 1.50 (1.24 – 1.80)
Dayan V [38]	2016	58	40,381	SAVR (*n*=39,568)	Any: 43.8%	Any PPM vs. none: HR 1.26 (1.16 – 1.36), *p*<0.0001
				TAVR (*n*=813)		Moderate vs. none: HR 1.01 (0.95 – 1.08), *p*=0.68
						Severe vs. none: HR 1.43 (1.14 – 1.80), *p*=0.002
						Severe vs. moderate: HR 1.33 (1.18 – 1.51), *p*<0.0001
Liao YB [48]	2017	30	4,691	TAVR only	Any: 33.0%	Any PPM vs. none (2 years): OR 0.98 (0.72 – 1.32)
					Moderate: 25.0%	Moderate vs. none (2 years): HR 1.05 (0.72 – 1.54)
					Severe: 11.0%	Severe vs. none (2 years): HR 1.95 (0.38 – 9.91)
He S [47]	2020	18	72,016	TAVR only	Any: 32.0%	Any PPM vs. none (2 years): OR 0.99 (0.79 – 1.24)
					Severe: 10.0%	
Lim OZH [46]	2022	22	1,15,442	TAVR only	Any: 31.0%	Any PPM vs. none (mean FU 2.0 years): HR 0.97 (0.83 – 1.13)
					Severe: 10.7%	Severe vs. none (mean FU 1.4 years): HR 1.86 (1.05 – 3.29), *p*=0.034
Sá MP [45]	2023	23	81,969	TAVR only	Any: 23.9%	Any PPM vs. none (5 years): HR 1.09 (1.04 – 1.14), *p*<0.001
					Moderate: 24.1%	Moderate vs. none (5 years): HR 1.03 (0.96 – 1.10), *p*=0.398
					Severe: 10.9%	Severe vs. none (5 years): HR 1.25 (1.16 – 1.36), *p*<0.001
Sá MP [39]	2024	65	1,22,989	SAVR only	Any: 55.6%	Any PPM vs. none (25 years): HR 1.16 (1.13 – 1.18), *p*<0.001
					Moderate: 51.4%	Moderate vs. none (20 years): HR 1.09 (1.06 – 1.11), *p*<0.001
					Severe: 9.3%	Severe vs. none (20 years): HR 1.29 (1.24 – 1.35), *p*<0.001

CI=confidence interval, HR=hazard ratio, OR=odds ratio, PPM=prosthesis patient mismatch, SAVR=surgical aortic valve replacement, TAVR=transcatheter aortic valve replacement

### Impact of measured PPM in TAVR

In TAVR studies the impact of PPM on clinical outcomes has mainly been assessed using measured PPM and results have varied (Table 5) [19,40–44]. Hermann HC et al. investigated the clinical impact of PPM from the STS/ACC TVT registry in 62,125 patients. At 1 year, there was no difference in mortality or heart failure hospitalization between patients with no PPM and moderate PPM, however, patients with severe PPM had a significantly higher rate of mortality or hospitalization compared to the other two groups [40].

**Table 5:** Selected studies reporting clinical outcomes of PPM after TAVR

First Author	Database	Year	*N* of patients	Type of THV	Type of PPM	Criteria of PPM	Incidence of PPM	FU	Mortality
Herrmann HC [40]	STS/ACC TVT registry	2018	62,125	BE, SE	Measured PPM	#1	Moderate: 25%	1 year	Moderate vs. none: 15.6% vs. 15.9% (adjusted HR 1.00, CI 0.93-1.07)
							Severe: 12%		Severe vs. none: 17.2% vs. 15.9% (adjusted HR 1.19, CI 1.09-1.31)
Tang GHL [41]	STS/ACC TVT registry	2021	42,174 (de novo)	SE	Measured PPM	#2	De novo cohort	1 year	De novo cohort
							Moderate: 13.9%		Severe vs. moderate or none: 13.8% vs. 12.7% (adjusted HR 1.00, CI 0.87-1.16)
			5,446 (TAV in SAV)				Severe: 5.3%		Moderate vs. none: 13.7% vs. 12.5%, *p*=0.04
									Severe vs. none: 13.8% vs. 12.5%, *p*=0.12
Ternacle J [19]	PARTNER 2	2021	936 (SAVR)	BE	Measured PPM	#2	SAVR cohort	5 years	SAVR cohort
							Moderate: 31%		Moderate vs. none: 40.8% vs. 33.1% (HR 1.27, CI 0.95-1.70), *p*=0.103
							Severe: 24%		Severe vs. none: 43.1% vs. 33.1% (HR 1.40, CI 1.03-1.91), *p*=0.03
			1,069 (TAVR))				TAVR cohort		TAVR cohort
							Moderate: 28%		Moderate vs. none: 40.8% vs. 36.2% (HR 1.14, CI 0.90-1.45), *p*=0.266
							Severe: 6%		Severe vs. none: 48.0% vs. 36.2% (HR 1.40, CI 0.92-2.14), *p*=0.116
					Predicted PPM	#2	SAVR cohort	5 years	SAVR cohort
							Moderate: 28%		Moderate vs. none: 43.7% vs. 40.3% (HR 1.11, CI 0.88-1.39), *p*=0.38
							Severe: 1%		Severe vs. none: 81.8% vs. 40.3% (HR 3.08, CI 1.58-5.99), *p*<0.01
							TAVR cohort		TAVR cohort
							Moderate: 21%		Moderate vs. none: 36.9% vs. 41.4% (HR 0.86, CI 0.67-1.10), *p*=0.22
							Severe: 0.1%		Severe vs. none: HR not available, *p*=0.465
Tomii D [42]	Swiss-TAVI registry	2023	2,463	BE, SE	Measured PPM	#2	Moderate: 27%	10 years	Moderate vs. none: 89.7% vs. 84.3% (adjusted HR 1.17, CI 1.00-1.38), *p*=0.055
							Severe: 9%	(median 429 days)	Severe vs. none: 94.6% vs. 84.3% (adjusted HR 1.19, CI 0.92-1.54), *p*=0.187
					Predicted PPM	#2	Moderate: 11%	10 years	Moderate vs. none: 82.0% vs. 86.8% (adjusted HR 0.73, CI 0.55-0.96), *p*=0.022
							Severe: 1%	(median 429 days)	Severe vs. none: 66.5% vs. 86.8% (adjusted HR 0.54, CI 0.24-1.22), *p*=0.139
Al-Azizi K [43]	Baylor Scott & White Health-Care System in Texas	2024	3,016	BE, SE	Measured PPM	#2	Moderate: 19%	5 years	Moderate vs. none: 45% vs. 47%, adjusted HR 1.05 (CI 0.88-1.26), *p*=0.59
							Severe: 6%		Severe vs. none: 54% vs. 47%, adjusted HR 1.26 (CI 0.96-1.66), *p*=0.095
					Predicted PPM	#2	Moderate: 21%	5 years	Moderate vs. none: 45% vs. 47%, adjusted HR 1.11 (CI 0.93-1.33), *p*=0.227
							Severe: 0.8%		Severe vs. none: 33% vs. 47%, adjusted HR 1.03 (CI 0.42-2.49), *p*=0.954
Guthoff H [44]	IMPACT TAVR registry	2024	18,477	BE, SE	Measured PPM	#2	Moderate: 21%	2 years	None: 18.5%, moderate: 18.6%, severe: 21.7%
							Severe: 4%		Severe vs. moderate or non: adjusted HR 1.14 (CI 0.94-1.38), *p*=0.186
					predicted PPM	#2	Moderate: 7%	4	None: 21.1%, moderate: 18.4%, severe: 17.2%
							Severe: 0.6%		Severe vs. moderate or non: adjusted HR 0.92, 95% CI 0.52-1.62, *p*=0.766

#1: none (>0.85 cm^2^/m^2^), moderate (0.85-0.65 cm^2^/m^2^), and severe (≤0.65 cm^2^/m^2^)

#2 (VARC3 criteria): If BMI is <30 kg/m^2^, none (>0.85 cm^2^/m^2^), moderate (0.85-0.66 cm^2^/m^2^), and severe (≤0.65 cm^2^/m^2^). If BMI is ≧30 kg/m2, none (>0.70 cm^2^/m^2^), moderate (0.70-0.55 cm^2^/m^2^), and severe (≤0.55 cm^2^/m^2^)

BE=balloon-expandable, CI=confidence interval, HR=hazard ratio, PPM=prosthesis patient mismatch, SAVR=surgical aortic valve replacement, SE=self-expanding, TAVR=transcatheter aortic valve replacement

A meta-analysis of reconstructed Kaplan-Meier curves (*n* = 81,969) reported that severe PPM, but not moderate PPM, was associated with higher mortality after TAVR compared with no PPM (Table 4) [45]. In another meta-analysis, only severe PPM after TAVR was associated with higher mortality, whereas any PPM was not [46,47].

On the contrary, there are studies reporting that PPM has no effect on mortality. Al-Azizi et al. investigated the impact of PPM in 3,016 patients undergoing TAVR and neither severe nor moderate PPM, irrespective of valve type, was associated with increased mortality for 5 years [43]. The IMPACT TAVR registry investigated the impact of measured and predicted PPM in TAVR patients. In 18,477 patients, severe measured PPM was associated with significantly higher mortality at 2-year follow-up compared to no PPM in unadjusted analyses. However, in a multivariable Cox analysis, severe measured PPM did not show a significant relationship with mortality compared to non-severe (i.e. no or moderate) measured PPM [44]. One meta-analysis also found no significant differences in either short- or mid-term mortality between patients with no-PPM and those with any PPM, moderate PPM or severe PPM [48].

Differences in patient demographics, as well as study designs, such as the number of patients, follow-up duration, completeness of follow-up rate, TAVR valves used, etc. may contribute to these conflicting results. A report from the STS/ACC TVT registry showed that severe PPM after self-expanding supra-annular THVs was not associated with increased 1-year mortality [41]. On the contrary, and as mentioned above, a report from the same registry, including balloon-expandable and self-expanding valves, showed a significant effect of severe PPM on mortality [40]. In the IMPACT TAVR registry, although not statistically significant, a marginal effect of severe PPM on mortality was observed after balloon-expandable THVs, whereas no such effect was observed after self-expanding THVs [44].

### Measured vs. predicted PPM in TAVR

Ternacle et al. used data from the PARTNER 2 study, and were the first to report the incidence and clinical impact of predicted PPM in TAVR (Table 5). Their study demonstrated that compared to measured PPM, the use of predicted PPM significantly reduced the incidence of PPM in both SAVR and TAVR. In TAVR patients, severe predicted PPM was nearly absent, with neither measured nor predicted PPM showing a significant association with mortality. However, in SAVR, severe predicted PPM was more strongly associated with increased mortality than severe measured PPM, suggesting that predicted PPM may be more prognostically relevant in the surgical setting [19].

The clinical impact of predicted PPM in TAVR remains uncertain and may be of limited value (Table 5) [42–44] Tomii et al. assessed the influence of measured and predicted PPM on long-term mortality using data from 2,463 patients in the Swiss-TAVI registry. Predicted PPM was associated with a lower incidence of PPM compared to measured PPM. Neither moderate nor severe measured PPM significantly affected 5- or 10-year mortality. Interestingly, moderate predicted PPM was paradoxically associated with lower mortality compared to no PPM, raising questions about the predictive validity of this approach [42]. Similarly, the IMPACT-TAVR study showed that the use of predicted PPM reduced the incidence of PPM, but neither measured nor predicted PPM was significantly associated with 2-year mortality [44].

### Differential clinical impact of PPM in SAVR versus TAVR

The negative impact of PPM on clinical outcomes has been consistently observed in SAVR, whereas findings in TAVR remain inconsistent. As discussed above, differences in the assessment of PPM (measured vs. predicted EOA), the inherent limitations of each method, and variations in PPM definitions make direct comparisons between SAVR and TAVR challenging. Nevertheless, in a meta-analysis of SAVR studies, PPM was consistently associated with worse outcomes even when assessed using measured EOA [39]. In contrast, in TAVR studies, the use of predicted PPM has not demonstrated differences in outcomes between patients with and without PPM. ^19,42–44^

From a procedural perspective, in SAVR, the prosthetic valve is surgically implanted with a sewing ring and is structurally fixed within the annulus, resulting in a relatively stable and well-defined effective orifice area. Therefore, PPM in SAVR more directly reflects a fixed mechanical limitation of forward flow, which may lead to a more consistent association with clinical outcomes. In contrast, TAVR prostheses are deployed within the native valve and are subject to variability in expansion, positioning, interaction with calcification, and procedural factors. As a result, the effective orifice area may be more influenced by multiple factors beyond prosthesis size alone, potentially attenuating or obscuring the relationship between PPM and outcomes.

Differences in patient characteristics may also partly explain this discrepancy. SAVR is more commonly performed in younger, more active patients, in whom the hemodynamic consequences of PPM may be more pronounced.

Methodological differences across studies may also play an important role. TAVR studies are typically restricted to patients with aortic stenosis, whereas SAVR studies may include patients with aortic regurgitation and those undergoing concomitant procedures, which could affect outcomes. Furthermore, the inclusion of periprocedural mortality – events that may not be directly attributable to PPM – can influence the observed associations. Importantly, studies using measured PPM inherently include only patients who survive to postoperative echocardiographic assessment, whereas studies using predicted PPM may include patients who die during or shortly after the procedure. This difference in patient inclusion may further contribute to the inconsistent associations observed in TAVR. Therefore, more standardized approaches are warranted to enable more consistent evaluation across studies.

## Call for alternatives to PPM? Fitting index

Considering the methodological limitations and the uncertain clinical implications of PPM, it may be time to consider alternative concepts for evaluating valve–patient interactions. Garcia D et al. proposed the concept of energy loss index (ELI) to account for the pressure recovery [49]. Energy loss reflects the loss of energy as blood flows from the left ventricle into the aorta across the aortic valve. In an in vitro experimental model, they demonstrated an inverse relationship between energy loss and the coefficient (EOA*Aa) / (Aa – EOA), where Aa denotes the cross-sectional area of the ascending aorta. When this coefficient was indexed to BSA, it was defined as the ELI. An ELI ≦ 0.52 cm^2^/m^2^ was associated with adverse clinical outcomes in subjects with moderate or severe aortic stenosis. However, the clinical implications of the energy loss index in TAVR patients remain debatable [50,51]. Another potential approach is post-TAVR CT assessment of THV geometry, including valve underexpansion and eccentricity, which may reflect device–annulus interaction more directly. Several studies have reported associations between CT-defined THV deformation and subsequent clinical outcomes [52,53]. Although post-TAVR CT provides detailed assessment of THV geometry, it is not routinely performed in many centers, limiting its widespread applicability. Alternatively, as described in the editorial by Ternacle J and Pibarot P on the PERIGON study, potential approaches include indexing EOA by fat free-mass instead of BSA, or the EOA indexed to the native aortic annulus area [54].

**Fig. 4:**
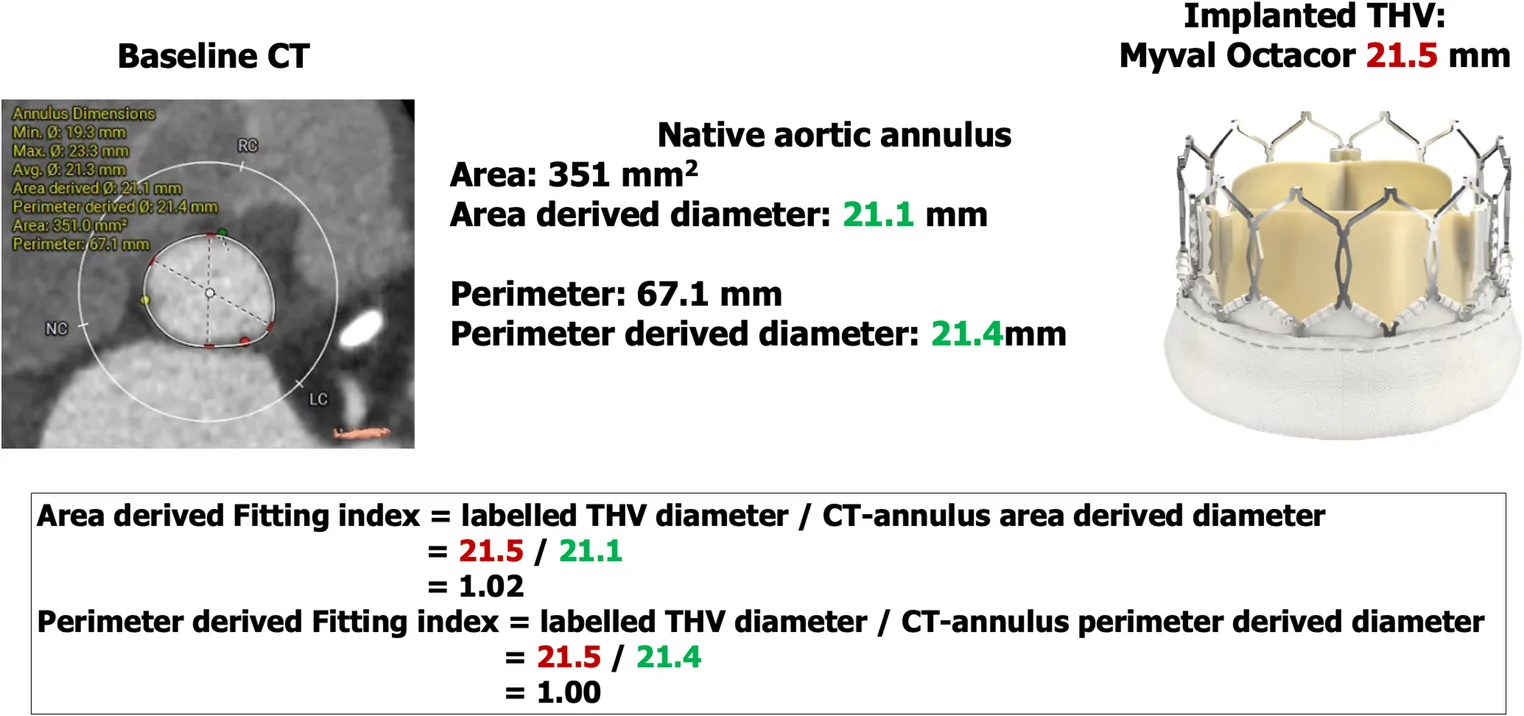
An example of calculating the Fitting Index. The area-derived and perimeter-derived diameters were calculated from the native aortic annulus area and perimeter measured on preprocedural CT, assuming a circular annulus. Area-derived diameter = 2$\:\sqrt{area/3.14}$. Perimeter-derived diameter=perimeter/3.14. CT=computed tomography, THV=transcatheter heart valve

In contemporary TAVR practice, preprocedural CT assessment is essential for valve sizing. Unlike SAVR, where annular enlargement is an option, in TAVR we are limited to selecting the type and size of THVs based on anatomical information. In this context, the “Fitting Index” may offer a practical and anatomically based metric. The Fitting Index is defined as the ratio between the THV diameter (valve size) and the aortic annulus diameter as measured by preprocedural CT. It reflects how well the selected valve fits within the annular anatomy. Area-derived Fitting index = (THV nominal diameter [THV size)] / aortic annulus diameter derived from aortic annulus area. Perimeter-derived Fitting index = (THV nominal diameter [THV size)] / aortic annulus diameter derived from aortic annulus perimeter (Fig. 4). Table 6 compares the fitting index with measured and predicted PPM.

**Table 6:** Features and comparisons of the fitting index and PPM

	Fitting index	Measured PPM	Predicted PPM
Formula	Aortic annulus area (preprocedural CT) derived diameter / implanted nominal THV diameter	EOA (directly measured) / BSA (cm^2^/m^2^)	EOA (based on reference publication) / BSA (cm^2^/m^2^)
	Aortic annulus perimeter (preprocedural CT) derived diameter / implanted nominal THV diameter		
Preprocedural prediction	Yes	No	Yes
Aortic annulus area (CT) is considered	Yes	No	No
Valve size is considered	Yes	No	No
Body size (BSA, BMI) is considered	No	Yes	Yes
Echocardiographic parameters	No	Yes	Yes

BMI=body mass index, BSA=body surface area, CT=computed tomography, EOA=effective orifice area, PPM=prosthesis patient mismatch, THV=transcatheter heart valve

**Fig. 5:**
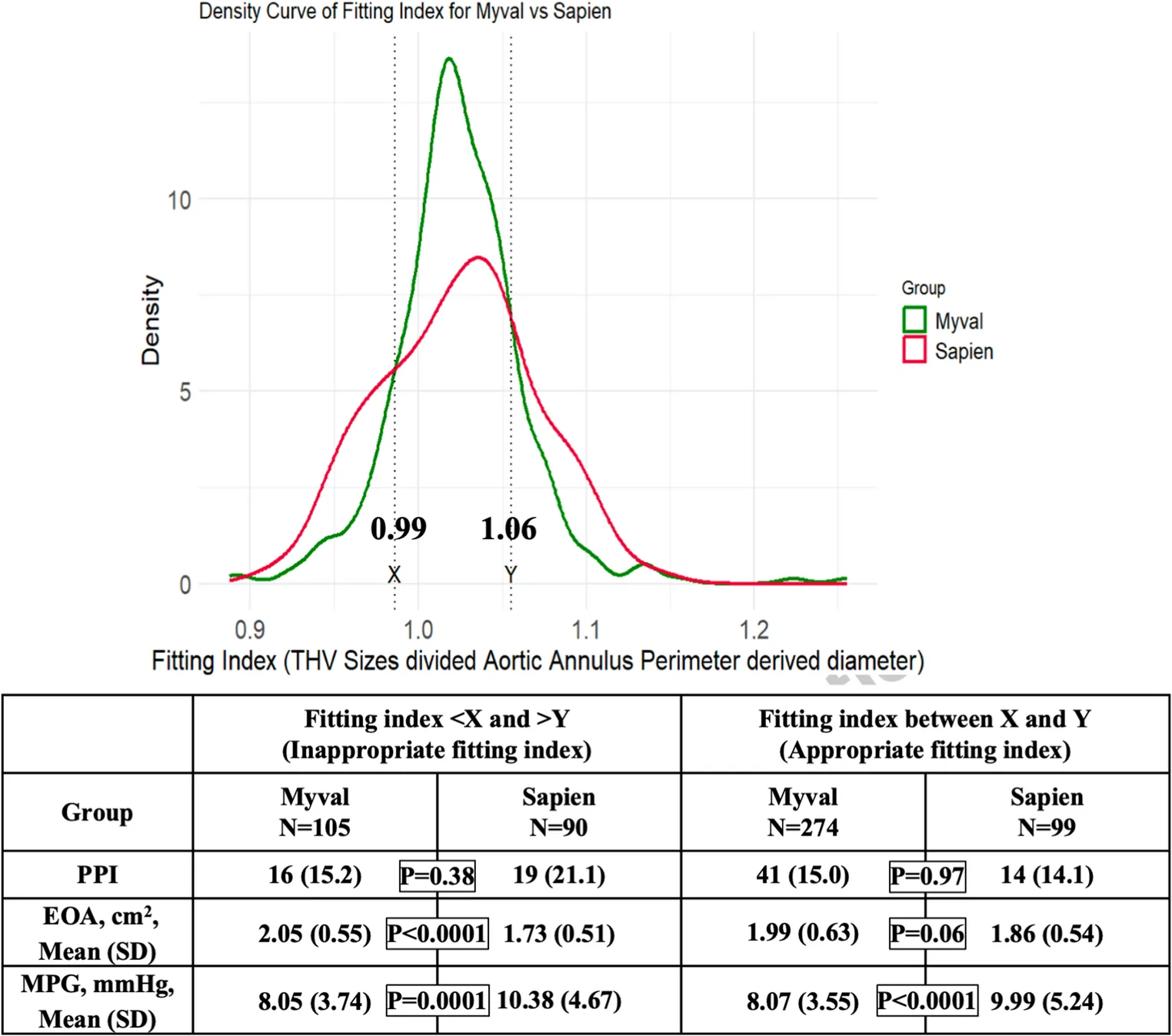
Fitting index and the incidence of new permanent pacemaker implantation, effective orifice area, and mean pressure gradient. Density curves of perimeter-derived fitting index in Myval series and Sapien series in the LANDMARK trial. Patients were divided based on the crossing points (< X or > Y: inappropriate fitting, > X or < Y: appropriate fitting; X = 0.99, Y = 1.06). Rates of new permanent pacemaker implantation, effective orifice area, and mean pressure gradient at 30 days are tabulated. The figure is adapted from van Royen N et al.^56^

We investigated the perimeter-derived Fitting Index in the Myval THV series (*n* = 368) and Sapien THV series (*n* = 195) in the Landmark trial [55-57], and Fig. 5 shows the density curve [56]. Notably, while the Myval has a narrow shape in the density curve, the Sapien has a wide shape. This difference relates to the availability in the Myval group of intermediate nominal sizes (21.5, 24.5, and 27.5 mm) as well as conventional nominal sizes (20, 23, 26, and 29 mm), which enables a better fit to the native aortic annulus.

We investigated the relationship between the fitting index and the incidence of new permanent pacemaker implantation (PPI), EOA, and mean pressure gradient at 30 days. The population was divided into two groups using the crossing points of the density curve of the fitting index (X = 0.99 and Y = 1.06): those with a fitting index less than X or greater than Y were categorized as inappropriate fitting, whilst those between X and Y were appropriate fitting (Fig. 5**and supplementary Fig. 2**). The better fit of the Myval series may help explain their observed larger EOA and lower mean pressure gradient.

A key limitation of this post hoc analysis is the lack of recorded data on THV balloon overfilling or underfilling, which hindered adjustment of the prosthesis area or perimeter dimensions. In addition, actual valve expansion was not evaluated by fluoroscopy or postprocedural CT. Furthermore, the Fitting Index is inherently dependent on the accuracy of annular sizing. The Fitting index was also not evaluated in the Evolut series, because the labeled valve sizes and corresponding aortic annulus area/perimeter substantially differ between self-expanding and balloon-expandable valves. This fundamental difference in sizing methodology makes direct comparison of the Fitting index across different valve platforms inappropriate. Even within balloon-expandable platforms, the relationship between labeled valve size and actual deployed valve dimensions may vary according to device-specific design characteristics, such as frame architecture, radial force, recoil behavior, and leaflet configuration. Another major limitation is the lack of validated outcomes for this method. Consequently, the clinical value of the broad size matrix of the Myval series—including both intermediate and conventional sizes—has not yet been established, and the findings related to the fitting index should be considered hypothesis-generating. Although the current sample size is limited, further insights are expected following a planned pooled analysis with the COMPARE-TAVI study (Myval THV series; *n* = 514, Sapien THV series; *n* = 517) [58].

## Summary

Almost half a century ago, cardiac surgeons introduced the concept of PPM using EOA indexed by BSA, a surrogate of cardiac output. Indeed, after SAVR, PPM has proven to be a robust predictor of vital status. With the advent of echocardiography, the EOA became assessable non-invasively; however, several cautions should be considered when interpreting the EOA and EOAi. Furthermore, invasive assessment of AVA using the Gorlin formula may overestimate AVA at low gradients, limiting its usefulness in reliably assessing PPM.

The impact of measured PPM post-TAVR on long-term mortality remains inconsistent across studies, with conflicting results. The clinical relevance of PPM may vary depending on valve design, and this requires further validation.

Predicted PPM in TAVR, while offering certain advantages over measured PPM, has not clearly demonstrated prognostic value and may have limited clinical usefulness. Given these limitations, we propose an alternative fitting index derived from data routinely collected during the mandatory pre-TAVR multi-slice CT. The clinical value of the fitting index warrants prospective investigation.

## Supplementary Information

Below is the link to the electronic supplementary material.

Supplementary Material 1

## References

[1] Serruys PW, Cribier A, Eltchaninoff H, Grube E, Makkar R, Onuma Y, Webb JG, Leon MB. Transcatheter Aortic Valve Implantation Current and Future Developments. 2 ed. Boca Raton: CRC; 2024.

[2] Rahimtoola SH. The problem of valve prosthesis-patient mismatch. Circulation. 1978;58:20–4. 10.1161/01.cir.58.1.20. 348341

[3] Baumgartner H, Hung J, Bermejo J, Chambers JB, Evangelista A, Griffin BP, Iung B, Otto CM, Pellikka PA, Quinones M et al. Echocardiographic assessment of valve stenosis: EAE/ASE recommendations for clinical practice. *J Am Soc Echocardiogr*. 2009;22:1–23; quiz 101–102. 10.1016/j.echo.2008.11.029 19130998

[4] Kim HJ, Park SJ, Koo HJ, Kang JW, Yang DH, Jung SH, Choo SJ, Chung CH, Lee JW, Kim JB. Determinants of effective orifice area in aortic valve replacement: anatomic and clinical factors. J Thorac Dis. 2020;12:1942–51. 10.21037/jtd-20-188. 32642097 PMC7330291

[5] Gorlin R, Gorlin SG. Hydraulic formula for calculation of the area of the stenotic mitral valve, other cardiac valves, and central circulatory shunts. I. Am Heart J. 1951;41:1–29. 10.1016/0002-8703(51)90002-6. 14799435

[6] Kappetein AP, Head SJ, Genereux P, Piazza N, van Mieghem NM, Blackstone EH, Brott TG, Cohen DJ, Cutlip DE, van Es GA, et al. Updated standardized endpoint definitions for transcatheter aortic valve implantation: the Valve Academic Research Consortium-2 consensus document. J Am Coll Cardiol. 2012;60:1438–54. 10.1016/j.jacc.2012.09.001. 23036636

[7] Pibarot P, Dumesnil JG. Valve prosthesis-patient mismatch, 1978 to 2011: from original concept to compelling evidence. J Am Coll Cardiol. 2012;60:1136–9. 10.1016/j.jacc.2012.07.005. 22995023

[8] Varc-3 Writing C, Genereux P, Piazza N, Alu MC, Nazif T, Hahn RT, Pibarot P, Bax JJ, Leipsic JA, Blanke P, et al. Valve Academic Research Consortium 3: updated endpoint definitions for aortic valve clinical research. Eur Heart J. 2021;42:1825–57. 10.1093/eurheartj/ehaa799. 33871579

[9] Son AY, Baldridge AS, Churyla A, Pham DT, Mehta CK, Johnston DR, McCarthy PM, Malaisrie SC. Trends and Outcomes of Aortic Root Enlargement During Bioprosthetic Aortic Valve Replacement. Ann Thorac Surg Short Rep. 2025;3:1–5. 10.1016/j.atssr.2024.09.007. 40098830 PMC11910798

[10] Eisenga JB, Pickering T, McCullough KA, Banwait J, Hale S, Harrington KB, Brinkman WT, Mack MJ, DiMaio JM, Schaffer JM. Surgeon Frequency of Aortic Root Enlargement and Long-Term Survival in Medicare Beneficiaries Undergoing Surgical Aortic Valve Replacement. Am J Cardiol. 2025;246:16–24. 10.1016/j.amjcard.2025.03.009. 40068783

[11] Durko AP, Pibarot P, Atluri P, Bapat V, Cameron DE, Casselman FPA, Chen EP, Dahle G, Elefteriades JA, Lancellotti P, et al. Essential Information on Surgical Heart Valve Characteristics for Optimal Valve Prosthesis Selection: Expert Consensus Document From the European Association for Cardio-Thoracic Surgery (EACTS)-The Society of Thoracic Surgeons (STS)-American Association for Thoracic Surgery (AATS) Valve Labelling Task Force. Ann Thorac Surg. 2021;111:314–26. 10.1016/j.athoracsur.2020.05.060. 33036738

[12] Bapat VN, Attia R, Thomas M. Effect of valve design on the stent internal diameter of a bioprosthetic valve: a concept of true internal diameter and its implications for the valve-in-valve procedure. JACC Cardiovasc Interv. 2014;7:115–27. 10.1016/j.jcin.2013.10.012. 24440016

[13] Hahn RT, Pibarot P. Prosthesis-patient mismatch in transcatheter and surgical aortic valve replacement. Ann Cardiothorac Surg. 2024;13:211–23. 10.21037/acs-2023-aae-0166. 38841078 PMC11148757

[14] Stinis CT, Abbas AE, Teirstein P, Makkar RR, Chung CJ, Iyer V, Genereux P, Kipperman RM, Harrison JK, Hughes GC, et al. Real-World Outcomes for the Fifth-Generation Balloon Expandable Transcatheter Heart Valve in the United States. JACC Cardiovasc Interv. 2024;17:1032–44. 10.1016/j.jcin.2024.02.015. 38456883

[15] Yamamoto M, Yashima F, Shirai S, Tada N, Naganuma T, Yamawaki M, Yamanaka F, Mizutani K, Noguchi M, Ueno H, et al. Performance and outcomes of the SAPIEN 3 Ultra RESILIA transcatheter heart valve in the OCEAN-TAVI registry. EuroIntervention. 2024;20:579–90. 10.4244/EIJ-D-23-00996. 38726714 PMC11067722

[16] Pibarot P, Herrmann HC, Wu C, Hahn RT, Otto CM, Abbas AE, Chambers J, Dweck MR, Leipsic JA, Simonato M, et al. Standardized Definitions for Bioprosthetic Valve Dysfunction Following Aortic or Mitral Valve Replacement: JACC State-of-the-Art Review. J Am Coll Cardiol. 2022;80:545–61. 10.1016/j.jacc.2022.06.002. 35902178

[17] Zoghbi WA, Jone PN, Chamsi-Pasha MA, Chen T, Collins KA, Desai MY, Grayburn P, Groves DW, Hahn RT, Little SH, et al. Guidelines for the Evaluation of Prosthetic Valve Function With Cardiovascular Imaging: A Report From the American Society of Echocardiography Developed in Collaboration With the Society for Cardiovascular Magnetic Resonance and the Society of Cardiovascular Computed Tomography. J Am Soc Echocardiogr. 2024;37:2–63. 10.1016/j.echo.2023.10.004. 38182282

[18] Fukui M, Garcia S, Lesser JR, Gossl M, Tang L, Caye D, Newell M, Hashimoto G, Lopes BBC, Stanberry LI, et al. Prosthesis-patient mismatch defined by cardiac computed tomography versus echocardiography after transcatheter aortic valve replacement. J Cardiovasc Comput Tomogr. 2021;15:403–11. 10.1016/j.jcct.2021.01.001. 33518457

[19] Ternacle J, Pibarot P, Herrmann HC, Kodali S, Leipsic J, Blanke P, Jaber W, Mack MJ, Clavel MA, Salaun E, et al. Prosthesis-Patient Mismatch After Aortic Valve Replacement in the PARTNER 2 Trial and Registry. JACC Cardiovasc Interv. 2021;14:1466–77. 10.1016/j.jcin.2021.03.069. 34238557

[20] Hahn RT, Leipsic J, Douglas PS, Jaber WA, Weissman NJ, Pibarot P, Blanke P, Oh JK. Comprehensive Echocardiographic Assessment of Normal Transcatheter Valve Function. JACC Cardiovasc Imaging. 2019;12:25–34. 10.1016/j.jcmg.2018.04.010. 29909110

[21] https://physics.stackexchange.com/questions/717442/pressure-recovery-what-is-it-and-why-do-we-want-it

[22] Abbas AE, Hanzel G, Shannon F, Gallagher M, Mando R, Chaddha A, Rodes-Cabau J, Pibarot P. Post-TAVR Trans-aortic Valve Gradients: Echocardiographic Versus Invasive Measurements: <em > The Role of Pressure Recovery</em >. Struct Heart. 2019;3:348–50. 10.1080/24748706.2019.1601798.

[23] Garcia D, Dumesnil JG, Durand LG, Kadem L, Pibarot P. Discrepancies between catheter and Doppler estimates of valve effective orifice area can be predicted from the pressure recovery phenomenon: practical implications with regard to quantification of aortic stenosis severity. J Am Coll Cardiol. 2003;41:435–42. 10.1016/s0735-1097(02)02764-x. 12575972

[24] Abbas AE, Khalili H, Madanat L, Elmariah S, Shannon F, Al-Azizi K, Waggoner T, Pilgrim T, Okuno T, Bavry A, et al. Echocardiographic Versus Invasive Aortic Valve Gradients in Different Clinical Scenarios. J Am Soc Echocardiogr. 2023;36:1302–14. 10.1016/j.echo.2023.06.016. 37507058

[25] Hatoum H, Hahn RT, Lilly S, Dasi LP. Differences in Pressure Recovery Between Balloon Expandable and Self-expandable Transcatheter Aortic Valves. Ann Biomed Eng. 2020;48:860–7. 10.1007/s10439-019-02425-8. 31792706 PMC11043825

[26] van den Dorpel MMP, Chatterjee S, Adrichem R, Verhemel S, Kardys I, Nuis RJ, Daemen J, Ren CB, Hirsch A, Geleijnse ML, et al. Prognostic value of invasive versus echocardiography-derived aortic gradient in patients undergoing TAVI. EuroIntervention. 2025;21:e411–25. 10.4244/EIJ-D-24-00341. 40259836 PMC11995293

[27] Rodes-Cabau J, Abbas AE, Serra V, Vilalta V, Nombela-Franco L, Regueiro A, Al-Azizi KM, Iskander A, Conradi L, Forcillo J, et al. Balloon- vs Self-Expanding Valve Systems for Failed Small Surgical Aortic Valve Bioprostheses. J Am Coll Cardiol. 2022;80:681–93. 10.1016/j.jacc.2022.05.005. 35597385

[28] Dumesnil JG, Yoganathan AP. Theoretical and practical differences between the Gorlin formula and the continuity equation for calculating aortic and mitral valve areas. Am J Cardiol. 1991;67:1268–72. 10.1016/0002-9149(91)90939-i. 2035453

[29] Abbas AE, Mando R, Kadri A, Khalili H, Hanzel G, Shannon F, Al-Azizi K, Waggoner T, Kassas S, Pilgrim T, et al. Comparison of Transvalvular Aortic Mean Gradients Obtained by Intraprocedural Echocardiography and Invasive Measurement in Balloon and Self-Expanding Transcatheter Valves. J Am Heart Assoc. 2021;10:e021014. 10.1161/JAHA.120.021014. 34585593 PMC8649128

[30] Vriesendorp MD, Groenwold RHH, Herrmann HC, Head SJ, De Lind RAF, Vriesendorp PA, Kappetein AP, Klautz RJM. The Clinical Implications of Body Surface Area as a Poor Proxy for Cardiac Output. Struct Heart. 2021;5:582–7. 10.1080/24748706.2021.1968089.

[31] Mannacio V, Mannacio L, Mango E, Antignano A, Mottola M, Caparrotti S, Musumeci F, Vosa C. Severe prosthesis-patient mismatch after aortic valve replacement for aortic stenosis: Analysis of risk factors for early and long-term mortality. J Cardiol. 2017;69:333–9. 10.1016/j.jjcc.2016.07.003. 27492659

[32] Fallon JM, DeSimone JP, Brennan JM, O’Brien S, Thibault DP, DiScipio AW, Pibarot P, Jacobs JP, Malenka DJ. The Incidence and Consequence of Prosthesis-Patient Mismatch After Surgical Aortic Valve Replacement. Ann Thorac Surg. 2018;106:14–22. 10.1016/j.athoracsur.2018.01.090. 29630873

[33] Dahlbacka S, Laakso T, Kinnunen EM, Moriyama N, Laine M, Virtanen M, Maaranen P, Ahvenvaara T, Tauriainen T, Husso A, et al. Patient-Prosthesis Mismatch Worsens Long-Term Survival: Insights From the FinnValve Registry. Ann Thorac Surg. 2021;111:1284–90. 10.1016/j.athoracsur.2020.06.026. 32805269

[34] Dismorr M, Glaser N, Franco-Cereceda A, Sartipy U. Effect of Prosthesis-Patient Mismatch on Long-Term Clinical Outcomes After Bioprosthetic Aortic Valve Replacement. J Am Coll Cardiol. 2023;81:964–75. 10.1016/j.jacc.2022.12.023. 36889875

[35] Head SJ, Mokhles MM, Osnabrugge RL, Pibarot P, Mack MJ, Takkenberg JJ, Bogers AJ, Kappetein AP. The impact of prosthesis-patient mismatch on long-term survival after aortic valve replacement: a systematic review and meta-analysis of 34 observational studies comprising 27 186 patients with 133 141 patient-years. Eur Heart J. 2012;33:1518–29. 10.1093/eurheartj/ehs003. 22408037

[36] Takagi H, Yamamoto H, Iwata K, Goto SN, Umemoto T. A meta-analysis of effects of prosthesis-patient mismatch after aortic valve replacement on late mortality. Int J Cardiol. 2012;159:150–4. 10.1016/j.ijcard.2012.04.084. 22584075

[37] Chen J, Lin Y, Kang B, Wang Z. Indexed effective orifice area is a significant predictor of higher mid- and long-term mortality rates following aortic valve replacement in patients with prosthesis-patient mismatch. Eur J Cardiothorac Surg. 2014;45:234–40. 10.1093/ejcts/ezt245. 23682010

[38] Dayan V, Vignolo G, Soca G, Paganini JJ, Brusich D, Pibarot P. Predictors and Outcomes of Prosthesis-Patient Mismatch After Aortic Valve Replacement. JACC Cardiovasc Imaging. 2016;9:924–33. 10.1016/j.jcmg.2015.10.026. 27236530

[39] Sa MP, Jacquemyn X, Van den Eynde J, Chu D, Serna-Gallegos D, Ebels T, Clavel MA, Pibarot P, Sultan I. Impact of Prosthesis-Patient Mismatch After Surgical Aortic Valve Replacement: Systematic Review and Meta-Analysis of Reconstructed Time-to-Event Data of 122 989 Patients With 592 952 Patient-Years. J Am Heart Assoc. 2024;13:e033176. 10.1161/JAHA.123.033176. 38533939 PMC11179750

[40] Herrmann HC, Daneshvar SA, Fonarow GC, Stebbins A, Vemulapalli S, Desai ND, Malenka DJ, Thourani VH, Rymer J, Kosinski AS. Prosthesis-Patient Mismatch in Patients Undergoing Transcatheter Aortic Valve Replacement: From the STS/ACC TVT Registry. J Am Coll Cardiol. 2018;72:2701–11. 10.1016/j.jacc.2018.09.001. 30257798

[41] Tang GHL, Sengupta A, Alexis SL, Bapat VN, Adams DH, Sharma SK, Kini AS, Kodali SK, Ramlawi B, Gada H, et al. Outcomes of Prosthesis-Patient Mismatch Following Supra-Annular Transcatheter Aortic Valve Replacement: From the STS/ACC TVT Registry. JACC Cardiovasc Interv. 2021;14:964–76. 10.1016/j.jcin.2021.03.040. 33958170

[42] Tomii D, Okuno T, Heg D, Nakase M, Lanz J, Praz F, Stortecky S, Reineke D, Windecker S, Pilgrim T. Long-term outcomes of measured and predicted prosthesis-patient mismatch following transcatheter aortic valve replacement. EuroIntervention. 2023;19:746–56. 10.4244/EIJ-D-23-00456. 37622754 PMC10654767

[43] Al-Azizi K, Moubarak G, Mohiuddin A, Szerlip M, Potluri S, Harrington K, Schaffer J, Brinkman W, Alsaid A, Wang Z, et al. Five-Year Outcomes of Measured and Predicted Prosthesis-Patient Mismatch following Transcatheter Aortic Valve Implantation. Am J Cardiol. 2024;231:11–9. 10.1016/j.amjcard.2024.08.013. 39209242

[44] Guthoff H, Abdel-Wahab M, Kim WK, Witberg G, Wienemann H, Thurow M, Shamekhi J, Eckel C, von der Heide I, Veulemans V, et al. Impact of Measured and Predicted Prosthesis-Patient Mismatch After Transcatheter Aortic Valve Replacement. JACC Cardiovasc Interv. 2024;17:2626–35. 10.1016/j.jcin.2024.08.041. 39603776

[45] Sa MP, Jacquemyn X, Van den Eynde J, Tasoudis P, Dokollari A, Torregrossa G, Sicouri S, Clavel MA, Pibarot P, Ramlawi B. Impact of Prosthesis-Patient Mismatch After Transcatheter Aortic Valve Replacement: Meta-Analysis of Kaplan-Meier-Derived Individual Patient Data. JACC Cardiovasc Imaging. 2023;16:298–310. 10.1016/j.jcmg.2022.07.013. 36648055

[46] Lim OZH, Mai AS, Ng CH, Tang A, Chin YH, Kong G, Ho YJ, Ong J, Tay E, Kuntjoro I, et al. Meta-Analysis Comparing Risk Factors, Incidence, and Outcomes of Patients With Versus Without Prosthesis-Patient Mismatch Following Transcatheter Aortic Valve Implantation. Am J Cardiol. 2022;170:91–9. 10.1016/j.amjcard.2022.01.023. 35193765

[47] He S, Fang Z. Incidence, predictors, and outcome of prosthesis-patient mismatch after transcatheter aortic valve replacement: A meta-analysis. Med (Baltim). 2020;99:e20717. 10.1097/MD.0000000000020717. PMC7302587 32541522

[48] Liao YB, Li YJ, Jun-Li L, Zhao ZG, Wei X, Tsauo JY, Xiong TY, Xu YN, Feng Y, Chen M, Incidence. Predictors and Outcome of Prosthesis-Patient Mismatch after Transcatheter Aortic Valve Replacement: a Systematic Review and Meta-analysis. Sci Rep. 2017;7:15014. 10.1038/s41598-017-15396-4. 29118326 PMC5678180

[49] Garcia D, Pibarot P, Dumesnil JG, Sakr F, Durand LG. Assessment of aortic valve stenosis severity: A new index based on the energy loss concept. Circulation. 2000;101:765–71. 10.1161/01.cir.101.7.765. 10683350

[50] Holy EW, Nguyen-Kim TDL, Hoffelner L, Stocker D, Stadler T, Stahli BE, Kebernik J, Maisano F, Kasel MA, Frauenfelder T, et al. Multimodality imaging derived energy loss index and outcome after transcatheter aortic valve replacement. Eur Heart J Cardiovasc Imaging. 2020;21:1092–102. 10.1093/ehjci/jeaa100. 32533142

[51] Okuno T, Pilgrim T, Heg D, Stortecky S, Praz F, Windecker S, Lanz J. Prosthesis-Patient Mismatch Based on Energy Loss Index After Transcatheter Aortic Valve Replacement. JACC Cardiovasc Interv. 2020;13:2584–6. 10.1016/j.jcin.2020.07.031. 33153575

[52] Fukui M, Bapat VN, Garcia S, Dworak MW, Hashimoto G, Sato H, Gossl M, Enriquez-Sarano M, Lesser JR, Cavalcante JL, et al. Deformation of Transcatheter Aortic Valve Prostheses: Implications for Hypoattenuating Leaflet Thickening and Clinical Outcomes. Circulation. 2022;146:480–93. 10.1161/CIRCULATIONAHA.121.058339. 35862182

[53] Nagasaka T, Patel V, Shechter A, Suruga K, Koren O, Chakravarty T, Cheng W, Ishii H, Jilaihawi H, Nakamura M, et al. Impact of Balloon-Expandable TAVR Valve Deformation and Calcium Distribution on Outcomes in Bicuspid Aortic Valve. JACC Cardiovasc Interv. 2024;17:2023–37. 10.1016/j.jcin.2024.07.018. 39260960

[54] Ternacle J, Pibarot P, Prosthesis-Patient, Mismatch. The Complex Interaction between Cardiac Output and Prosthetic Valve Effective Orifice Area. Struct Heart. 2021;5:588–90. 10.1080/24748706.2021.2010848.

[55] Baumbach A, van Royen N, Amat-Santos IJ, Hudec M, Bunc M, Ijsselmuiden A, Laanmets P, Unic D, Merkely B, Hermanides RS, et al. LANDMARK comparison of early outcomes of newer-generation Myval transcatheter heart valve series with contemporary valves (Sapien and Evolut) in real-world individuals with severe symptomatic native aortic stenosis: a randomised non-inferiority trial. Lancet. 2024. 10.1016/S0140-6736(24)00821-3. 38795719

[56] Royen Nv, Amat-Santos IJ, Hudec M, Bunc M, Ijsselmuiden A, Laanmets P, Unic D, Merkely B, Hermanides RS, Ninios V et al. Early outcomes of the novel Myval THV series compared to SAPIEN THV series and Evolut THV series in individuals with severe aortic stenosis. EuroIntervention e-e 10.4244/eij-d-24-00951 PMC11729642 39589296

[57] Serruys PW, Tobe A, Royen Nv, Amat-Santos IJ, Hudec M, Bunc M, Branden BJLVd, Laanmets P, Unic D, Merkely B et al. One-year outcomes of novel balloon-expandable versus contemporary transcatheter heart valves in severe aortic stenosis: the LANDMARK trial. *JACC*.0. doi: 10.1016/j.jacc.2025.10.076 41384893

[58] Terkelsen CJ, Freeman P, Dahl JS, Thim T, Norgaard BL, Mogensen NSB, Tang M, Eftekhari A, Povlsen JA, Poulsen SH, et al. SAPIEN 3 versus Myval transcatheter heart valves for transcatheter aortic valve implantation (COMPARE-TAVI 1): a multicentre, randomised, non-inferiority trial. Lancet. 2025. 10.1016/S0140-6736(25)00106-0. 40187364

