# Supplementary material for: Understanding prosthesis patient mismatch in aortic valve replacement: from pitfalls to clinical impact
Source: Cardiovasc Interv Ther. 2026 Aug 7;41(4):916–30. doi: 10.1007/s12928-026-01331-w (PMC13582111; doi:10.1007/s12928-026-01331-w)
Supplement: Supplementary file 1 [file 12928_2026_1331_MOESM1_ESM.pdf]

Supplementary materials

Supplementary Figure 1. Gorlin formula AVA vs mean pressure gradient

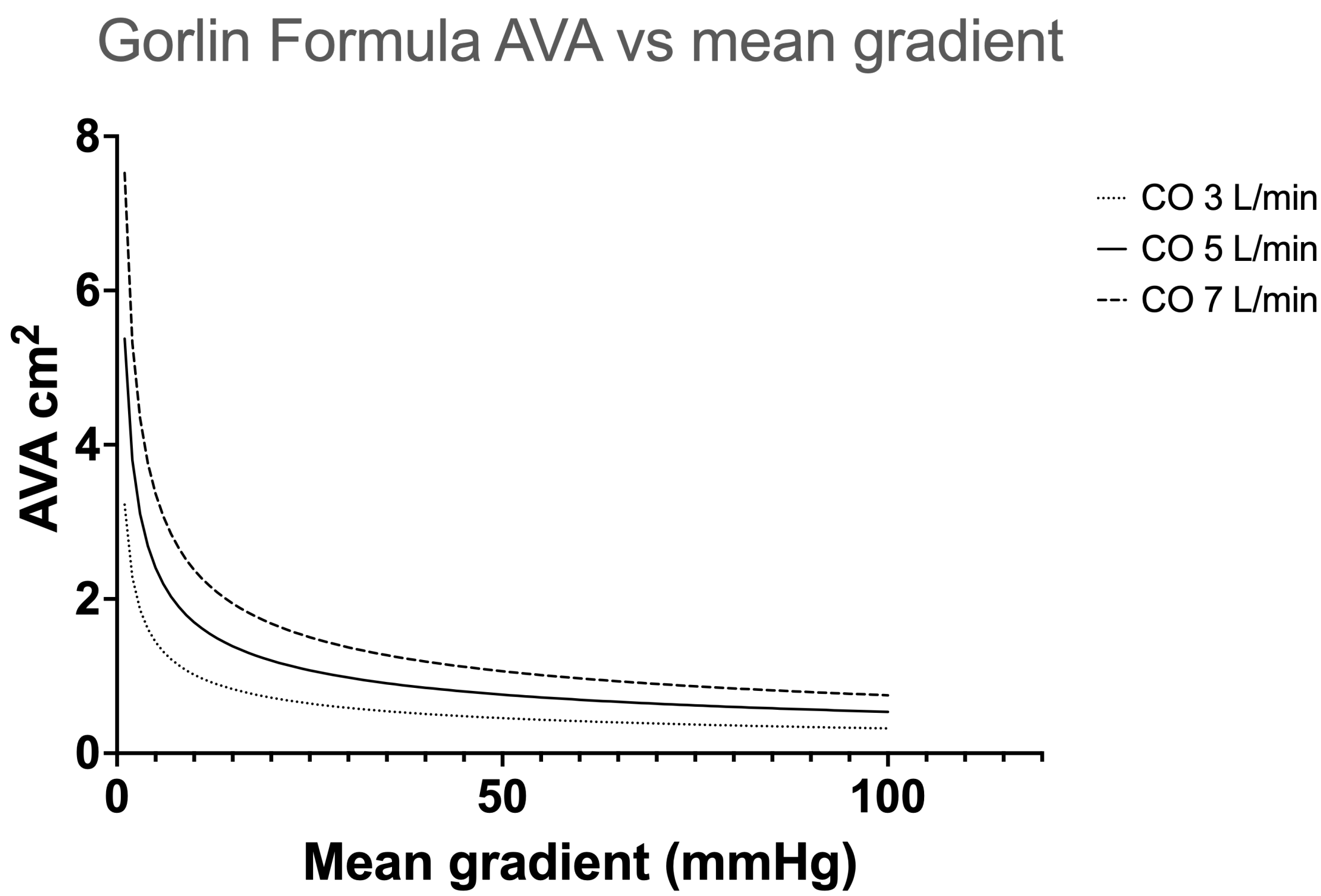

Relationship between mean pressure gradient and aortic valve area (AVA) calculated using the Gorlin formula at three cardiac output (CO) levels (3, 5, and 7 L/min), with heart rate (70 bpm) and ejection time (300 ms) held constant.

Supplementary Figure 2. Concept of the Fitting Index and its potential role in procedural planning

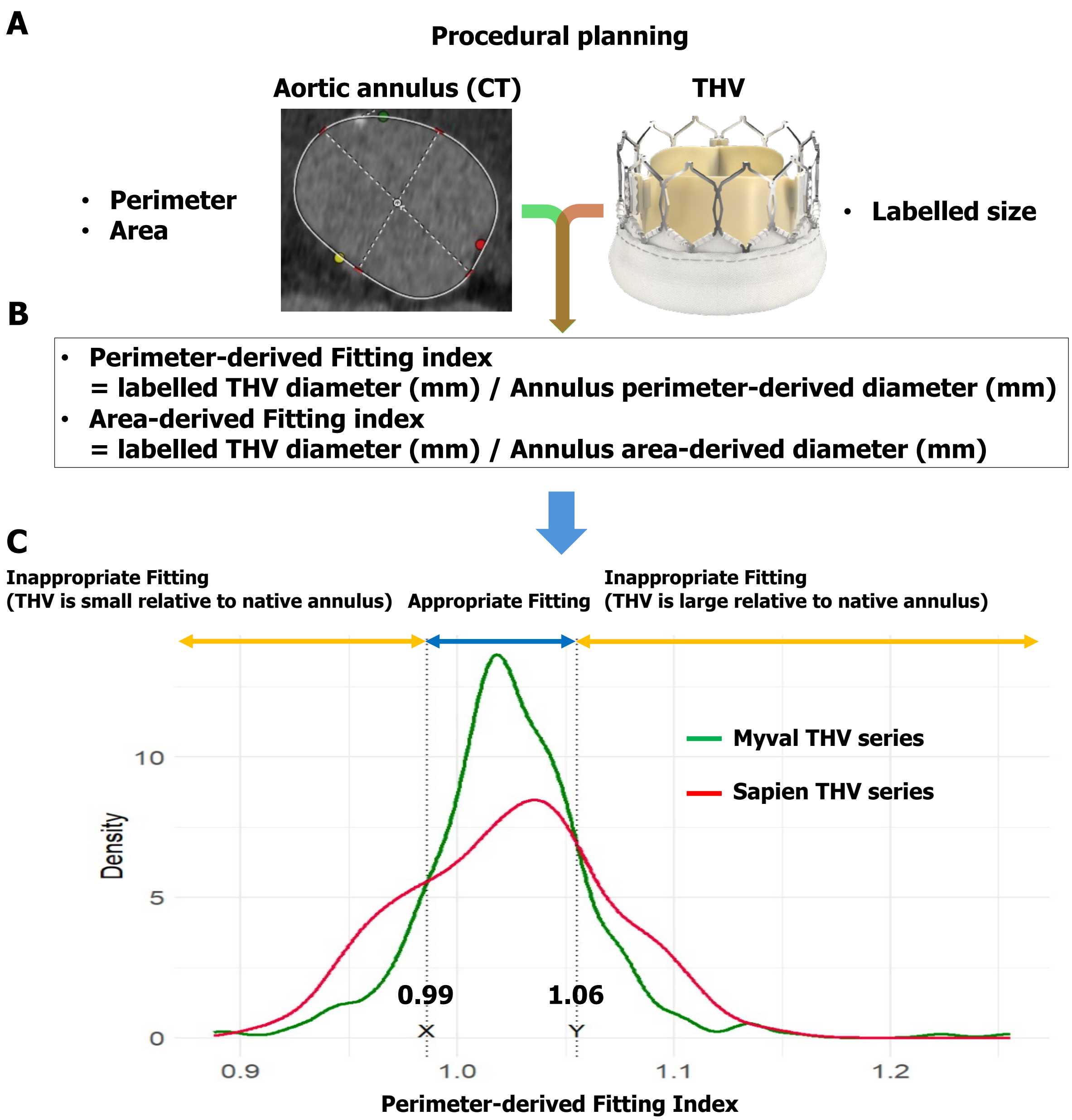

A: The Fitting Index can be calculated using preprocedural computed tomography and the labeled size of the THV which is going to be implanted. B: Perimeter-derived index=THV diameter/annulus perimeter-derived diameter. Area-derived Fitting index=THV diameter/annulus area-derived diameter. Perimeter- or area-derived diameters are calculated assuming that the

aortic annulus is circular. C: Density curve of the fitting index in the Myval THV series and Sapien THV series from the LANDMARK trial. The crossing points of X (=0.99) and Y (=1.06) may be potential thresholds for appropriate fitting. The clinical prognostic value of the fitting index needs to be validated in future studies. The figure is adapted from van Royen N et al[1].

1 Royen Nv, Amat-Santos IJ, Hudec M, Bunc M, Ijsselmuiden A, Laanmets P, Unic D, Merkely B, Hermanides RS, Ninios V, et al. Early outcomes of the novel Myval THV series compared to SAPIEN THV series and Evolut THV series in individuals with severe aortic stenosis. *EuroIntervention*.e-e. doi: 10.4244/eij-d-24-00951
